# Childhood wayfinding experience explains sex and individual differences in adult wayfinding strategy and anxiety

**DOI:** 10.1186/s41235-020-00220-x

**Published:** 2020-03-17

**Authors:** Vanessa Vieites, Shannon M. Pruden, Bethany C. Reeb-Sutherland

**Affiliations:** Department of Psychology, Florida International University, 11200 S.W. 8th Street, Miami, FL 33199 USA

**Keywords:** Wayfinding, Wayfinding strategies, Wayfinding anxiety, General anxiety, Sex differences, Mediation, Navigation, Spatial cognition

## Abstract

**Background:**

Anyone who has ever found themselves lost while driving in an unfamiliar neighborhood or forgotten where they parked their car can appreciate the importance of being able to navigate their environment. Navigation, or wayfinding, is a large-scale spatial ability that involves keeping track of the relative positions of objects and features in space, which allows for determining the path to a goal location. Early experiences shape spatial skill development, and research finds sex differences in spatial behaviors from preschool through adulthood, with males consistently outperforming females. The basis for sex differences in spatial aptitude is still debated, but explanations include differences in childhood spatial experience, the use of strategies for solving large-scale spatial problems, and spatial anxiety. The current study seeks to understand childhood wayfinding factors that may influence sex and individual differences in wayfinding strategies and wayfinding anxiety in adulthood.

**Method:**

One hundred fifty-nine undergraduate psychology students reported their childhood wayfinding experience (i.e., time spent outside, distance traveled), current use of wayfinding strategies (i.e., route strategy, orientation strategy), and current wayfinding anxiety and general anxiety levels.

**Results:**

Independent samples *t* tests revealed that, compared with females, males reported spending more time outside and traveling farther distances as children, having less current wayfinding anxiety and route strategy use, and having more current orientation strategy use. Mediation analyses found that distance traveled, but not time spent outdoors, during childhood mediated sex differences in route strategy use and wayfinding anxiety in adults, even when controlling for general anxiety. Furthermore, when controlling for participant sex and general anxiety, current wayfinding anxiety mediated the relationship between distance traveled during childhood and route strategy use in adults.

**Conclusion:**

The current findings provide potential environmental explanations for sex and individual differences in large-scale spatial behaviors, including wayfinding. Specifically, sex differences in early wayfinding experience may explain why males and females develop different strategies for navigating and different levels of wayfinding anxiety. Furthermore, regardless of sex, allowing children to explore and navigate their outdoor environments away from home may help lessen their fears about navigating and, in turn, improve the strategies they choose to traverse unfamiliar territories.

## Significance

Navigation, or wayfinding, is a highly adaptive, large-scale spatial ability that has several real-world implications for mobile animals. As humans, we use wayfinding skills when we visualize the layout of our neighborhoods, take shortcuts to work, and navigate an unfamiliar city. Individuals may navigate by route information, paying attention to which way to turn at certain landmarks, by orientation information, using cardinal directions and global reference cues to guide them, or both. Females show a preference for using the former strategy, while males tend to use the latter. Individuals who rely primarily on a route strategy to navigate are less efficient at doing so than those who rely mainly on an orientation strategy, possibly because route information is less reliable than orientation information. In turn, individuals who use a relatively inefficient strategy to navigate may have higher levels of wayfinding anxiety, which is also more prevalent in females than in males. Early wayfinding experiences have the potential to impact later wayfinding-related behaviors and apprehension, with males being allowed to roam farther than females during childhood. Given these considerations, we explored whether adults who spent more time outdoors and traveled farther distances as children now use one wayfinding strategy more than the other and have lower amounts of wayfinding anxiety, as well as which type of childhood wayfinding experience explains sex differences in wayfinding strategies and anxiety.

## Introduction

Navigating the space around us requires that we understand how to mentally represent our three-dimensional environment and the objects and features within it. This large-scale visuospatial ability, also referred to as “wayfinding” (Lawton, 2010), is highly adaptive and useful to mobile species (Newcombe, Uttal, & Sauter, 2013). For example, many species rely on their navigational abilities to search for their basic needs (e.g., food, water, shelter), find mates, and rejoin conspecifics after being separated from them (Newcombe et al., 2013). However, navigation not only has evolutionary significance for the survival of our species but also has practical, everyday importance for us as individuals. For humans, the ability to navigate has allowed us not only to understand our position in space so that we can traverse our surroundings but also to create symbolic, visual representations of the landscape in the form of navigational aids such as maps, compasses, and, more recently, the Global Positioning System (GPS; Newcombe et al., 2013). Without our wayfinding skills, we would quite literally be lost.

Importantly, early experiences shape spatial skill development, and children who participate in spatial activities (e.g., block play, environmental exploration, athletics) tend to perform better on tests of small-scale spatial abilities (i.e., designing blocks, locating embedded figures (Baenninger & Newcombe, 1989), Mental Rotation Test (Nazareth, Herrera, & Pruden, 2013)) and large-scale spatial abilities (i.e., navigation, mapmaking (Schug, 2016a)). From an early age, boys are found to engage more than girls in a variety of spatial activities (Schug, 2016a), and males consistently outperform females on tests that require them to mentally rotate and translate objects, from preschool (Lauer, Yhang, & Lourenco, 2019; Levine, Huttenlocher, Taylor, & Langrock, 1999) well into adulthood (Linn & Petersen, 1985). With the exception of mental rotation, reported sex differences in spatial skills are typically small (Nazareth, Huang, Voyer, & Newcombe, 2019) and/or do not emerge until after puberty (Voyer, Voyer, & Bryden, 1995). While the basis for sex differences in spatial cognition is still debated, recent explanations suggest that males have more spatial experience (i.e., wayfinding experience) than females do (Lawton & Kallai, 2002; Schug, 2016b), that different strategies for solving spatial problems may contribute to sex differences (Lawton, 1994, 1996; Nazareth, Killick, Dick, & Pruden, 2019; Stieff, Dixon, Ryu, Kumi, & Hegarty, 2014), and that wayfinding-specific spatial anxiety is related to both perceived and actual navigation ability (Lyons et al., 2018). In the current study, we examined the relationships between self-reported childhood wayfinding experience, the use of different wayfinding strategies, and wayfinding anxiety with the goal of explaining sex and individual differences. Our results shed light on which factors are potentially worth pursuing in experimental, longitudinal, and intervention studies to reduce sex and individual differences in spatial ability.

### Of maps and men: what we know about sex differences in large-scale spatial ability

It is often joked that women cannot read maps and that men refuse to ask for directions when lost, but is there truth to these popular stereotypes? Indeed, research consistently finds that, compared with females, males display several differences in wayfinding behaviors (Lawton, 2010). While sex differences are not typically found in young children (3–6-year-olds) on spatial reorientation tests involving geometric cues and a single landmark (e.g., Learmonth, Newcombe, Sheridan, & Jones, 2008; Vieites, Pruden, Shusterman, & Reeb-Sutherland, 2020), studies assessing prepubescent children (8–10-year-olds) have shown that males are quicker than females at finding a hidden platform in a virtual Morris water maze task (Newhouse, Newhouse, & Astur, 2007) and that males make fewer errors than females when tasked with relearning a maze in the absence of previously available landmarks (Jansen-Osmann & Wiedenbauer, 2004). Furthermore, preadolescent boys draw broader and more detailed maps of their home neighborhoods (Webley, 1981) and routes to school (Mathews, 1987) than similarly aged girls do, although these sex differences in mapmaking have been eliminated when controlling for children’s home ranges (Schug, 2016a).

Sex differences in wayfinding skills persist into adolescence and adulthood (Lawton, 2010). Whether in real-world (Malinowski & Gillespie, 2001) or virtual (Astur, Ortiz, & Sutherland, 1998; Cánovas, Espínola, Iribarne, & Cimadevilla, 2008) environments, males tend to be more efficient at navigating than females, requiring shorter travel times and being better able to find shortcuts to arrive at target locations. For example, in one study assessing spatial perception abilities in adolescence, eighth-grade boys were better able than girls to locate the position of a target figure on a two-dimensional contour map after studying that same target on a 360-degree virtual reality panorama of images (Park, Carter, Butler, Slykhuis, & Reid-Griffin, 2008). Likewise, compared with females, males are more accurate at reading maps and perform better when required to use maps to succeed at wayfinding tasks (Lawton, 2010). Taken together, these findings suggest that there are sex differences in large-scale spatial abilities favoring males, but that these differences typically emerge in late childhood, likely as a result of males’ greater experience with the spatial world.

### It’s a great big world: on early wayfinding experience

Some scholars have speculated that more time spent thinking about the spatial world could drive the development of spatial skills (Pruden, Levine, & Huttenlocher, 2011; Pruden, Nazareth, Odean, Abad, & Bravo, 2019). From early childhood, males show more of an interest than females in spatial toys (e.g., blocks, trucks)—those that are designed to be built, manipulated, and/or moved through space—and activities (e.g., rough-and-tumble play, sports). Play of this nature may facilitate spatial thinking (Cherney & Voyer, 2010; Connor & Serbin, 1977; Schug, 2016a). For example, studies suggest that environmental exploration can enhance visuospatial abilities (e.g., Nerlove, Munroe, & Munroe, 1971). In turn, other studies have shown that having high spatial aptitude leads to greater participation in spatial activities through self-selection (e.g., Newcombe & Dubas, 1992). In other words, individuals may gravitate toward activities they have previously excelled at and can perform while exerting minimal effort. Thus, there is likely a bidirectional relationship between experience and ability.

Although children are more restricted from wandering off alone today than in past decades (e.g., Prezza, 2007), across time periods and cultures, parents have allowed their sons to travel farther distances alone or with other children than they have their daughters. Moreover, boys’ chores often require them to travel farther from home than do girls’ chores (Schug, 2016a, 2016b). Males’ greater autonomy outside the home, reinforced by the sexual division of labor (White & Brinkerhoff, 2019; Wood & Eagly, 2002), presents boys with ample opportunities to practice spatial thinking while growing up. As such, males’ spatial competence and confidence may be enhanced relative to that of females. However, several studies have shown that when girls are given the same opportunities to navigate as boys are, they perform equally well on large-scale spatial tasks (Mathews, 1987; Munroe & Munroe, 1971; Webley, 1981), suggesting that wayfinding experience plays an important role in reducing sex differences in wayfinding abilities.

To date, few studies have examined later outcomes of having large-scale spatial experiences in childhood. In the current study, we examined adults’ childhood wayfinding experience in retrospect. While not longitudinal, this study can help us gauge whether childhood experiences shape wayfinding behaviors (i.e., wayfinding strategy use and wayfinding anxiety) later in life. For example, some research suggests that childhood wayfinding experience may predict the kinds of approaches adults use to navigate (Lawton & Kallai, 2002; Schug, 2016b), but these relationships have been understudied and, thus, remain unclear.

### To go north two miles or drive straight ahead until the stop sign: on wayfinding strategies

Individuals vary in the ways they successfully navigate, using a combination of approaches that include environmental features and cognitive representations of the space they are in (Shelton, Marchette, & Furman, 2013). Two popular types of strategies for wayfinding, also called “navigational style” (Schug, 2016b), are rooted in route knowledge and orientation (i.e., survey) knowledge (Marchette, Bakker, & Shelton, 2011; O’Keefe & Nadel, 1978; Russell & Ward, 1982; Shelton et al., 2013). On the one hand, individuals who prefer a route strategy to navigate tend to rely on information from salient landmarks (e.g., stop sign, grocery store) and use their own bodily movements (i.e., left, right, ahead) to find their way around unfamiliar territories. On the other hand, individuals who prefer to navigate using an orientation strategy tend to rely on cardinal directions (i.e., north, south, east, west) and keep track of their body’s position in relation to global reference points (e.g., the position of the sun).

Route information is typically more readily accessible and/or visible than global reference points, so it may be used more often by individuals who have difficulty “surveying” their environment. Meanwhile, orientation or survey knowledge may help individuals calculate shorter routes to their desired destinations (Boone, Maghen, & Hegarty, 2019). However, because landmarks and relational directions (i.e., left, right) are typically less stable than global cues, a route strategy may be less efficient (albeit still useful) in helping one navigate than an orientation strategy. Furthermore, because individuals who tend to use the less efficient route strategy may take longer to arrive at their goal destinations, they may become increasingly insecure about their navigation abilities and, thus, restrict themselves from further exploring unfamiliar places alone. However, individuals who display heightened anxiety about getting lost may prefer to use route information to guide their navigation because of its relative accessibility.

Research suggests there are sex differences in the strategies that males and females use on large-scale spatial tasks. For example, when giving or discussing directions, boys as young as 9 years old refer to distances and cardinal directions more often than do girls, who, in turn, begin to refer to landmarks and relative terms (e.g., left, right) more often than do boys around the age of 12 (Choi & Silverman, 2003). Adults also differ in the strategies by which they navigate. Males are more likely than females to report that they orient themselves by global cues (e.g., compass directions; Charleston, 2008), whereas females say more often than males that they travel by paying attention to landmarks and where to turn left or right (Lawton, 1994, 1996; Lawton & Kallai, 2002; Schug, 2016b). These findings are consistent with how males and females offer and follow directions. When giving directions, males tend to refer to cardinal directions and Euclidean (i.e., geometric) information such as exact distances, while females are inclined to mention environmental information such as landmarks (Lawton, 2001; Ward, Newcombe, & Overton, 1986). When following directions, females have an easier time navigating by environmental rather than Euclidean information, whereas males display the reverse tendency (Saucier et al., 2002). Taken together, these findings indicate that there are consistent sex differences in large-scale spatial strategies, with females often using the less efficient route strategy to navigate.

### Explaining sex differences in large-scale spatial ability: on wayfinding anxiety

Finally, there is reason to believe that wayfinding anxiety partially explains sex and individual differences in navigational ability and may be related to the navigation strategies males and females use. High levels of anxiety, or feelings of apprehension (Lauer, Esposito, & Bauer, 2018), can be debilitating and impair performance on cognitive tests (Hund & Minarik, 2006; Lauer et al., 2018; Lyons et al., 2018; Vytal, Cornwell, Arkin, Letkiewicz, & Grillon, 2013), and females display higher levels of general anxiety than do males (McLean & Anderson, 2009). However, anxiety can also be domain-specific, occurring only in certain situations or when performing certain kinds of tasks (e.g., math/spatial tests; Lauer et al., 2018). One domain-specific type of anxiety is spatial anxiety, which is triggered by tasks that require spatial reasoning (e.g., mental rotation, spatial perception; Lawton, 1994). Operational definitions of spatial anxiety include “worry about becoming lost” (Schmitz, 1999), anxious arousal (e.g., physiological changes in heart rate; Heller, Nitschke, Etienne, & Miller, 1997), and anxious apprehension (e.g., awareness of physiological changes, worry, and rumination; Heller et al., 1997) brought on by having to perform spatial tasks. The current study examines self-reported apprehension regarding wayfinding.

Like general anxiety, wayfinding anxiety is consistently found to be higher in females than in males (Lawton, 1994, 1996; Lawton & Kallai, 2002; Schug, 2016b). Because of real or perceived safety threats, females have a higher tendency than males to behave with caution in order to avoid harm when they go out into the world, which leads them to roam closer to familiar places (e.g., home) when they travel (Gagnon, Cashdan, Stefanucci, & Creem-Regehr, 2016). Females’ heightened awareness of threats to their personal safety, then, may increase their worries about navigating novel environments alone (Lawton & Kallai, 2002) and, in turn, may explain reported sex differences in navigation styles and memory for spatial locations (Gagnon et al., 2018). Furthermore, wayfinding anxiety has been linked to the use of an orientation strategy, whereby individuals with lower levels of wayfinding anxiety report greater use of an orientation strategy when navigating compared with individuals with higher levels of wayfinding anxiety (Hund & Minarik, 2006; Lawton, 1996), even when controlling for general anxiety (Lawton & Kallai, 2002). This may partially explain why, compared with females, males are more comfortable using orientation-based information when navigating. Lawton and Kallai (2002) also found that individuals in a combined sample from the United States and Hungary who reported more wayfinding anxiety also reported greater use of a route strategy. However, to our knowledge, no other study has yet reported a link, if any, between wayfinding anxiety and use of a route strategy*—*a less efficient navigational strategy than an orientation strategy—when navigating. Moreover, studies of this kind have inconsistently taken sex differences in general anxiety into account. Thus, it is unknown whether wayfinding anxiety is related to both relatively efficient and inefficient strategies for wayfinding and, if so, whether these results hold regardless of general anxiety.

### Current study

In the current study, we explored the role of childhood wayfinding experience in explaining sex and individual differences in both wayfinding strategies and wayfinding anxiety. This builds on previous research conducted by Lawton and Kallai (2002) and Schug (2016b), in which sex differences were found in adults’ childhood wayfinding experience, current wayfinding strategies (orientation and route strategy), and current wayfinding anxiety. Lawton and Kallai (2002) tested whether early wayfinding experience mediated sex differences in both wayfinding strategy and wayfinding anxiety and found that early wayfinding experience did not explain sex differences in wayfinding strategy (specifically, orientation strategy use) or wayfinding anxiety. Schug (2016b), on the other hand, tested whether use of an orientation strategy mediated the relationship between early wayfinding experience and later wayfinding anxiety but found no evidence for mediation. However, in their analyses, neither study reported investigating the two different types of early wayfinding experiences (i.e., distance traveled and time spent outside) to determine whether one type of experience was more meaningful than the other in explaining sex or individual differences in wayfinding strategy and anxiety. In addition, neither study reportedly examined the use of a route strategy in their mediation analyses, which we found peculiar because determining predictors and outcomes of less successful strategies for navigating is arguably as important as those of more efficient strategies. Thus, we decided to include both types of strategies as variables of interest in the current study.

Given that early experiences influence the development of spatial skills (Pruden et al., 2019; Schug, 2016a), the current study sought to assess the relationship between childhood wayfinding experiences and adult cognitive processes and affect associated with wayfinding. Our first two aims sought to unpack reported sex differences in wayfinding experience, strategy, and affect. Our last two aims sought to explore, more broadly, individual differences in these factors by holding participant sex constant in the analyses. Specifically, we aimed to address the following questions:

1. Are there sex differences in reported early childhood wayfinding experience, wayfinding strategies, and wayfinding anxiety?
2. Are sex differences in reported wayfinding strategies and wayfinding anxiety explained by different childhood wayfinding experiences?
3. Can childhood wayfinding experience predict the strategies individuals will develop for wayfinding and who will become anxious when navigating later in life?
4. Does reported wayfinding strategy explain individual differences in the relationship between childhood wayfinding experience and wayfinding anxiety?

Our first set of hypotheses concerned sex differences in childhood wayfinding experiences, wayfinding strategies, and wayfinding anxiety (Aim 1). On the basis of results of the studies by Lawton and Kallai (2002) and Schug (2016b), we predicted that males would report traveling farther distances and spending more time outdoors between the ages of 6 and 15 years, more frequent use of an orientation strategy and lower use of a route strategy when navigating, and lower levels of wayfinding anxiety than females.

In our second set of hypotheses, we examined childhood wayfinding experience as a potential mediator explaining sex differences in wayfinding strategies and wayfinding anxiety (Aim 2). Because Lawton and Kallai (2002) found that childhood wayfinding experience was related to both participant sex and orientation strategy use, we first hypothesized that participants’ childhood wayfinding experience would mediate the relationship between participant sex and current wayfinding strategy. Critically, we explored both types of wayfinding strategies (orientation and route strategy) in our mediation analyses. Second, we hypothesized that participants’ wayfinding anxiety would mediate the relationship between participant sex and wayfinding strategy. Last, because Lawton and Kallai (2002) found a link between adults’ early wayfinding experience and current wayfinding anxiety, we tested whether participants’ childhood wayfinding experience would mediate the relationship between participant sex and wayfinding anxiety. Importantly, when assessing wayfinding anxiety, we controlled for trait (i.e., general) anxiety because females tend to have higher levels of general anxiety than males (McLean & Anderson, 2009).

Third, we made predictions concerning individual differences in the relationship between childhood wayfinding experience and current wayfinding strategy and wayfinding anxiety. We predicted that individuals, regardless of participant sex, who had more experience navigating as children would report greater use of an orientation strategy and lower levels of anxiety when navigating (Aim 3).

Last, we examined potential mediators contributing to individual differences, regardless of participant sex, in the relationship between childhood wayfinding experience, wayfinding anxiety, and wayfinding strategy (Aim 4). Schug (2016b) proposed that a specific wayfinding strategy, specifically orientation strategy use, might mediate the relationship between childhood wayfinding experience and wayfinding anxiety in adulthood. We also hypothesized that wayfinding anxiety might instead mediate the relationship between childhood wayfinding experience and participants’ current wayfinding strategy use. While speculative, it is possible that having more (or less) exploratory wayfinding behavior during childhood may lead individuals, regardless of sex, to adopt an orientation-based (or route-based) style of wayfinding through the development of lower (or higher) levels of wayfinding anxiety. On the other hand, childhood exploration may reduce (or increase) later wayfinding anxiety through the development of an orientation-based (or route-based) wayfinding strategy. However, the specifics and directionality of these relationships remain inconclusive, so we tested them in Aim 4 by first focusing on wayfinding strategy as a potential mediator and then on wayfinding anxiety as a potential mediator in a reverse causal mediation analysis.

## Methods

### Participants

The sample consisted of 159 undergraduate students (85 females, *M*_*age*_ = 21.90; range = 18.04–35.73 years; *SD* = 3.39; 74 males, *M*_*age*_ = 23.09; range = 8.20–37.44 years; *SD =* 4.58) recruited from introductory psychology courses at a large, public R1 university in the southeastern United States. One hundred fifty-four participants provided their birth dates (*M*_*age*_ = 22.45 years; range = 18.03–37.44 years; *SD* = 4.01). There was no statistically significant difference between the ages of males and females, *t*(152) =1.854; *p* = .066. The sample represented the ethnic and racial composition of the surrounding county. Regarding ethnicity, 72.2% of our sample identified as Hispanic or Latino. Regarding race, 70.4% identified as white, 16.4% as black or African American, 2.5% as Asian, 8.8% as another race, 0.6% as Native Hawaiian or Pacific Islander, and 0.6% as Native American or Alaskan Native. Furthermore, 9.5% of our sample identified as white non-Hispanic. One participant did not provide information on racial identity.

### Procedure

Participants were asked to complete four questionnaires during an in-person visit to the laboratory. All questionnaires were administered via paper and pencil. In addition, demographic data, including participant sex, race, and ethnicity, were collected. The following questionnaires were given: Childhood Wayfinding Experience Questionnaire (Lawton & Kallai, 2002), Wayfinding Strategies Questionnaire (Lawton, 1996; Lawton & Kallai, 2002), Wayfinding Anxiety Questionnaire (Lawton & Kallai, 2002), and State-Trait Anxiety Inventory (STAI; Spielberger, 1989). The order of questionnaires was fixed with anxiety questionnaires presented last to ensure that we did not inadvertently increase or elicit anxiety before completion of experience and strategy questionnaires.

### Materials

#### Childhood wayfinding experience

This eight-item questionnaire (Lawton & Kallai, 2002) asked participants how often they were allowed to go out on errands and how far they were allowed to travel by themselves or with friends on foot, skates, or bicycle at each of four different age ranges: 6–7, 8–10, 11–13, and 14–15 years. For the question on how often they were allowed to go outside as children, participants were given five time options: almost never, 1–3 times/year, 4–11 times/year, 1–3 times/month, once/week, or more often. For the question on how far they were allowed to travel as children, participants were given five distance options: less than 1/4 mile (1/2 km), 1/2 mile (1 km), 1–2 miles (2–3 km), 3–4 miles (4–7 km), 5 miles (8 km), or farther. An average score (out of 5) was computed and used as a dependent measure for each component (i.e., time spent outdoors, distance traveled) of childhood wayfinding experience, with higher scores indicating more time outdoors and farther distances traveled during childhood. The original scale’s Cronbach alpha, or internal reliability, was found to be .85 (Lawton & Kallai, 2002).

#### Wayfinding strategies

This 17-item cross-cultural questionnaire (Lawton & Kallai, 2002) asked participants about their use of two kinds of navigational strategies: an orientation strategy and a route strategy. The former strategy involves using global reference points (e.g., the sun, cardinal directions), whereas the latter strategy involves keeping track of landmarks and one’s left–right bodily movements to find one’s way around. Because not all participants may belong to cultures in which cars and other forms of private transportation are common, Lawton and Kallai (2002) replaced all references to driving in the original questionnaire (Lawton, 1994, 1996) with broader references to traveling. For each item, participants were asked to rank on a scale of 1 (not at all true) to 5 (very true) the extent to which they used the strategy described in the given scenario (e.g., I kept track of the direction [north, south, east, or west] in which I was going; clearly visible signs pointing the way to different sections of the building or complex were important to me) when navigating. An average score (out of 5) was computed and used as a dependent measure for each type of navigational strategy (i.e., orientation strategy, route strategy), with higher scores indicating greater use of that particular strategy. Lawton and Kallai (2002) found that Cronbach’s alpha was .79 for the 11 orientation strategy questions and .70 for the 6 route strategy questions.

#### Wayfinding anxiety

This eight-item cross-cultural questionnaire (Lawton & Kallai, 2002) asked participants to rate on a scale of 1 (not at all anxious) to 5 (severely anxious) how anxious they were when navigating in a variety of scenarios (e.g., finding my way to an appointment in an unfamiliar area of a city or town; trying a new route that I think will be a shortcut, without a map). To accommodate participants of different cultures, Lawton and Kallai (2002) replaced all references to driving with broader references to traveling. An average score (out of 5) was computed and used as a dependent measure (i.e., wayfinding anxiety), with higher scores indicating higher levels of anxiety about navigating. Cronbach’s alpha was .87 (Lawton & Kallai, 2002).

#### General anxiety

The 40-item STAI (Spielberger, 1989) asked participants on a scale of 1 (not at all) to 4 (very much so), how they felt at that moment (i.e., their state anxiety) and how they generally felt (i.e., their trait anxiety). Because there tend to be observed sex differences in general anxiety (Altemus, Sarvaiya, & Neill Epperson, 2014), we only assessed the 20 trait items to control for general anxiety. Trait anxiety items included “I lack self-confidence” and “I am calm, cool, and collected.” We computed an average score (out of 4) for the trait anxiety scale, with higher scores indicating higher levels of trait anxiety. Alpha coefficients for this scale have ranged from .86 to .95 (Spielberger, Gorsuch, Lushene, Vagg, & Jacobs, 1983). Because we sought to control for general anxiety, we used trait anxiety scores as a covariate any time we analyzed wayfinding anxiety.

## Results

Prior to data analyses, we assessed for outliers, defined as data points that were located above or below 3 standard deviations from the mean. In total, eight outliers were identified and removed from further data analyses: three from the childhood wayfinding questionnaire, four from the orientation strategy questionnaire, and one from the route strategy questionnaire. Table 1 demonstrates the bivariate correlations between time spent outdoors, distance traveled, route strategy, orientation strategy, wayfinding anxiety, and trait anxiety.

**Table 1:** Pearson correlation with time spent outdoors, distance traveled, route strategy, orientation strategy, wayfinding anxiety, and trait anxiety

	1	2	3	4	5	6
1. Time spent outdoors	*–*					
2. Distance traveled	.58***	*–*				
3. Route strategy	−.15**	−.28**	*–*			
4. Orientation strategy	−.02	−.017	.23**	*–*		
5. Wayfinding anxiety	−.13	−.24**	.29***	−.16*	*–*	
6. Trait anxiety	−.02	−.15	.18*	.012	.37***	*–*

*p ≤ .05, **p ≤ .01, ***p ≤ .001

### Aim 1: Are there sex differences in reported early childhood wayfinding experience, wayfinding strategies, and wayfinding anxiety?

**Fig. 1:**
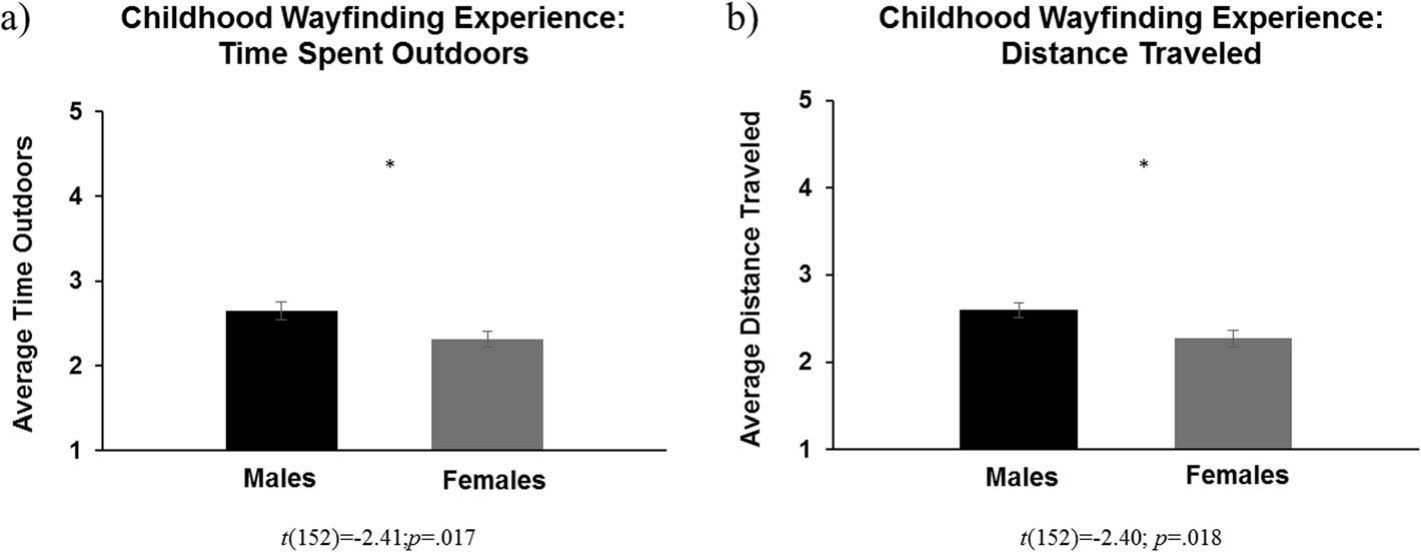
Sex difference in time spent outdoors (**a**) and distance traveled (**b**) between 6 and 15 years of age. *p < .05

**Fig. 2:**
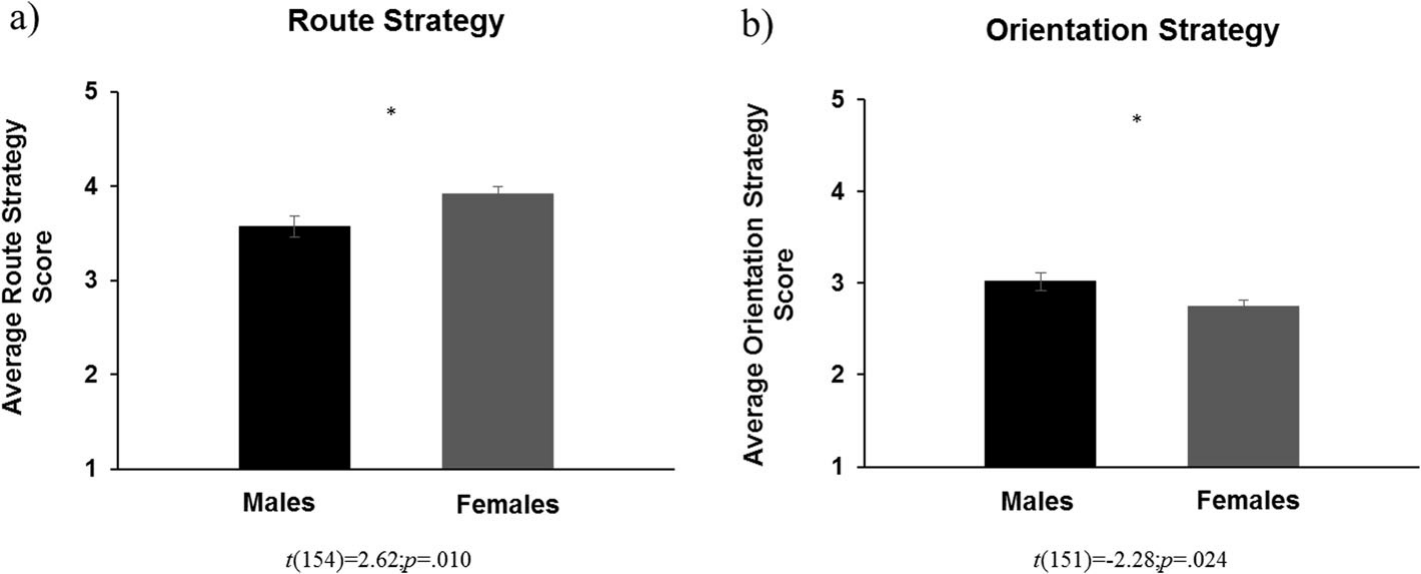
Sex difference in route strategy (**a**) and orientation strategy (**b**) use. *p < .05

**Fig. 3:**
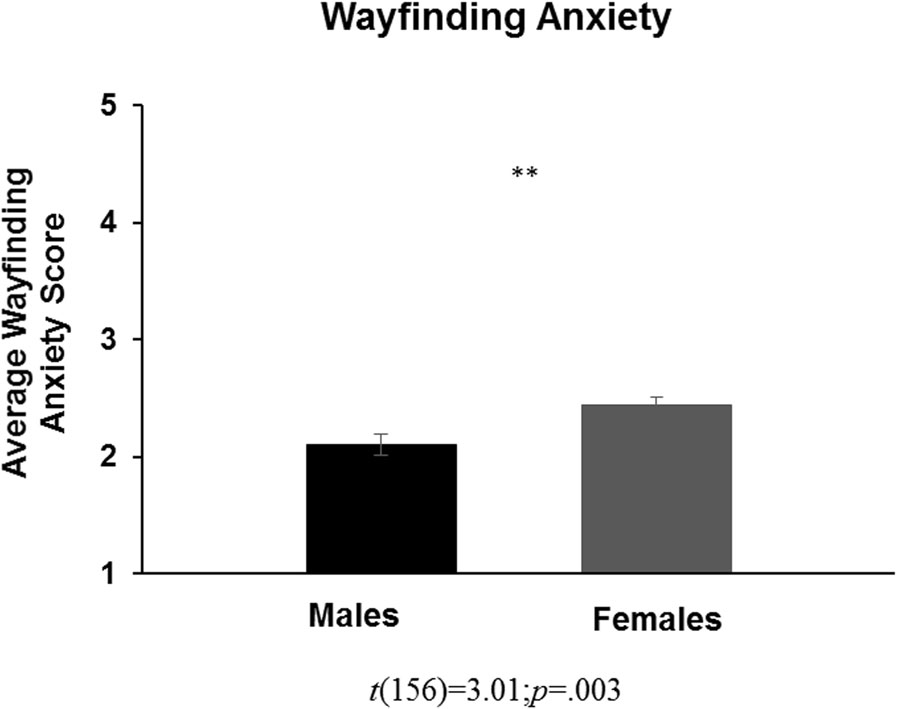
Sex difference in wayfinding anxiety. **p < .01

Independent samples *t* tests revealed that, compared with females, males reported being allowed to spend more time outdoors, *t*(152) = − 2.41; *p* = .017, *d* = 0.39 (Fig. 1a), and to travel farther distances, *t*(152) = − 2.40; *p* = .018, *d* = 0.39 (Fig. 1b), between the ages of 6 and 15 years. Males also used more of an orientation strategy when navigating, *t*(151) = − 2.28; *p* = .024, *d* = 0.36 (Fig. 2b), used less of a route strategy when navigating, *t*(154) = 2.62; *p* = .010, *d* = 0.42 (Fig. 2a), and had less wayfinding anxiety, *t*(156) = 3.01; *p* = .003, *d* = 0.48 (Fig. 3), than females. Paired-samples *t* tests, collapsed across sex, determined that participants reported using a route strategy significantly more often than an orientation strategy when navigating, *t*(151) =10.77; *p* < .001, *d* = 0.87. This was found for both males, *t*(70) =4.49; *p* < .001, *d* = 0.53, and females *t*(80) =11.69; *p* < .001, *d* = 1.30. Table 2 demonstrates the descriptive statistics for each variable, separated by participant sex.

**Table 2:** Means and standard deviations of the variables by participant sex

Variable	Females		Males		All			
	M (SD)	*n*	M (SD)	*n*	M (SD)	*n*	Minimum	Maximum
Total childhood wayfinding experience	2.30 (0.80)	84	2.65 (0.73)	71	2.46 (0.79)	155	1.00	4.63
Distance traveled	2.27 (0.91)	84	2.59 (0.75)	70	2.42 (0.85)	154	1.00	4.75
Time spent outdoors	2.31 (0.83)	84	2.65 (0.89)	70	2.46 (0.87)	154	1.00	4.75
Route strategy preference	3.92 (0.75)	85	3.57 (0.91)	71	3.76 (0.84)	156	1.33	5.00
Orientation strategy preference	2.75 (0.60)	81	3.02 (0.84)	72	2.46 (0.87)	153	1.00	4.82
Wayfinding anxiety	2.44 (0.65)	85	2.10 (0.76)	73	2.28 (0.72)	158	1.00	3.88
Trait anxiety	2.01 (0.54)	84	1.89 (0.54)	73	1.96 (0.54)	157	1.00	3.30

*Note.* Sex was dummy coded as 0 for females and 1 for males

### Aim 2: Are sex differences in reported wayfinding strategies and wayfinding anxiety explained by different childhood wayfinding experiences?

Because there were significant sex differences in childhood wayfinding experience, wayfinding strategies, and wayfinding anxiety, we hypothesized that sex differences in participants’ wayfinding strategies and anxiety might be mediated by having a specific type of childhood wayfinding experience.

#### Wayfinding strategy

Using the PROCESS macro (version 2.16.3; Model 4; Hayes, 2009) in IBM SPSS Statistics version 20 software (IBM Corp., Armonk, NY) to conduct mediation analyses, we first examined whether total childhood wayfinding experience (and in separate analyses, distance traveled and time spent outdoors) mediated the relationship between participant sex and wayfinding strategy use. We report in the main text results related to route strategy use because none of the analyses using orientation strategy use reached statistical significance (see Supplementary Materials, Supplemental Figure 1a-c, for a report of these null findings).

**Fig. 4:**
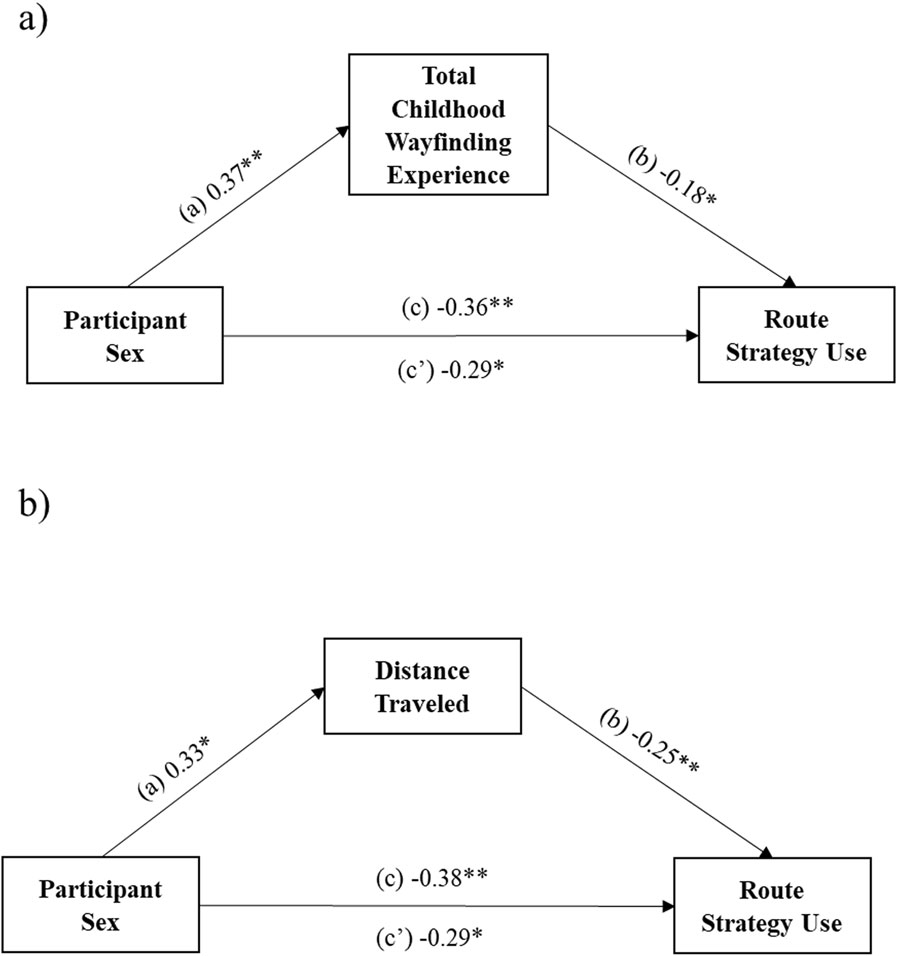
Direct and indirect effects of participant sex on route strategy use through the mediators total childhood wayfinding experience (**a**) and distance traveled during childhood (**b**). *p < .05, **p < .01, ***p < .001

Total childhood wayfinding experience (distance traveled plus time spent outdoors) partially mediated the sex difference in route strategy use (see Fig. 4a for mediation model). The total effect of participant sex on route strategy use was significant, path *c* = − 0.36; *t*(151) = − 2.66; *p* = .009; 95% CI, − 0.63, − 0.09, indicating that males’ route strategy scores were 0.36 units lower than those of females, when not controlling for the effect of the mediator total childhood wayfinding experience. The effect of participant sex on childhood wayfinding experience (path *a* = 0.37, *t*[151] = 2.30, *p* = .003, 95% CI [0.13, 0.62]), and, in turn, the effect of total childhood wayfinding experience on route strategy use when controlling for participant sex (path *b* = − 0.18, *t*[150] = − 2.06, *p* = .042, 95% CI [− 0.35, − 0.01]) were both significant. Last, the direct effect of participant sex on route strategy use when controlling for the mediator remained statistically significant, path *c′* = − 0.29, *t*(150) = − 2.13, *p* = .035, 95% CI [− 0.56, − 0.02]. Because females were dummy coded as 0 and males as 1, the negative coefficient of the direct effect indicates that males’ route strategy scores were 0.29 units lower than those of females when controlling for total childhood wayfinding experience.

We next explored whether it was a specific type of wayfinding experience (distance traveled or time spent outdoors) that mediated the reported relationship above. Regarding type of wayfinding experience, distance traveled during childhood partially mediated the sex difference in route strategy use (see Fig. 4b for mediation model). Time spent outdoors did not reach statistical significance, so we focused only on the mediation finding for distance traveled in the main text (see Supplementary Materials, Supplemental Figure 2, for null findings related to time spent outdoors). The total effect of participant sex on route strategy use was statistically significant, path *c* = − 0.38, *t*(150) = − 2.78, *p* = .006, 95% CI [− 0.64, − 0.11], indicating that males’ route strategy scores were 0.38 units lower than those of females when the effect of the mediator, distance traveled, was left unaltered. The effect of participant sex on distance traveled was significant, path *a* = 0.33, *t*(150) = 2.42, *p* = .017, 95% CI [0.06, 0.60]. The effect of distance traveled on route strategy use when controlling for participant sex was also statistically significant, path *b* = − 0.25, *t*(149) = − 3.14, *p* = .002, 95% CI [− 0.40, − 0.09]. Finally, the direct effect of participant sex on route strategy use when controlling for distance traveled remained statistically significant, path *c′* = − 0.29, *t*(149) = − 2.20, *p* = .030, 95% CI [− 0.56, − 0.03]. The negative coefficient of the direct effect indicates that males’ route strategy scores were 0.29 units lower than those of females when controlling for the mediator. However, this mediation was no longer found when controlling for wayfinding anxiety (see Supplementary Materials, Supplemental Figure 3, for null findings related to distance traveled, controlling for wayfinding anxiety), suggesting that wayfinding anxiety might be an important factor in explaining sex differences in wayfinding strategy preferences.

#### Wayfinding anxiety

**Fig. 5:**
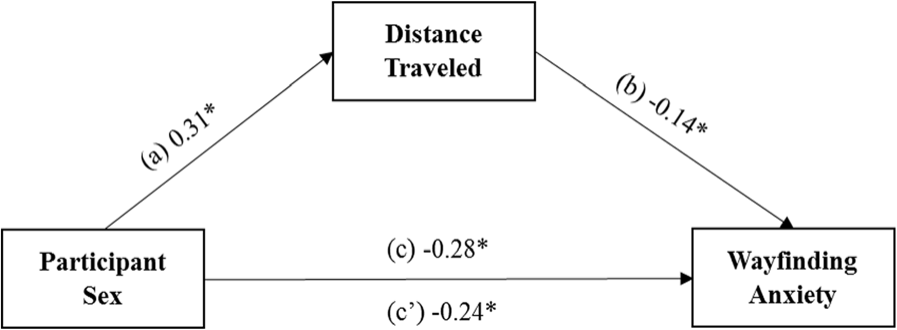
Direct and indirect effects of participant sex on wayfinding anxiety through the mediator distance traveled, controlling for trait anxiety. *p < .05, **p < .01, ***p < .001

We tested the hypothesis that total childhood wayfinding experience might mediate the sex difference in wayfinding anxiety, while controlling for trait anxiety. We found no mediation with our mediator variable as total childhood wayfinding experience (see Supplementary Materials, Supplemental Figure 4a, for null findings related to total childhood wayfinding experience). However, when we conducted further analyses assessing type of wayfinding experience, we found that distance traveled (but not time spent outdoors; see Supplementary Materials, Supplemental Figure 4b, for null findings related to time spent outdoors) partially mediated the sex difference in wayfinding anxiety, even when controlling for trait anxiety (see Fig. 5 for mediation model). The total effect of participant sex on wayfinding anxiety was statistically significant, controlling for trait anxiety, path *c* = − 0.28, *t*(150) = − 2.60, *p* = .010, 95% CI [− 0.49, − 0.07]. Male wayfinding anxiety scores were 0.28 units lower than those of females, when not controlling for the mediator, distance traveled. Participant sex was significantly related to distance traveled, when controlling for trait anxiety, path *a* = 0.31, *t*(150) = 2.25, *p* = .026, 95% CI [0.04, 0.58]. The effect of distance traveled on wayfinding anxiety, when controlling for participant sex and trait anxiety, was also statistically significant, path *b* = − 0.14, *t*(149) = − 2.12, *p* = .036, 95% CI [− 0.26, − 0.01]. The direct effect of participant sex on wayfinding anxiety, controlling for distance traveled and trait anxiety, remained statistically significant, path *c′* = − 0.24, *t*(149) = − 2.21, *p* = .029, 95% CI [− 0.45, − 0.03]. Male wayfinding anxiety scores were 0.24 units lower than those of females, when controlling for the mediator and covariate. Mediation analyses were corrected for multiple comparisons using a false discovery rate (FDR) procedure (Benjamini & Hochberg, 1995).

### Aim 3: Can childhood wayfinding experience predict the strategies individuals will develop for wayfinding and who will become anxious when navigating later in life?

**Fig. 6:**
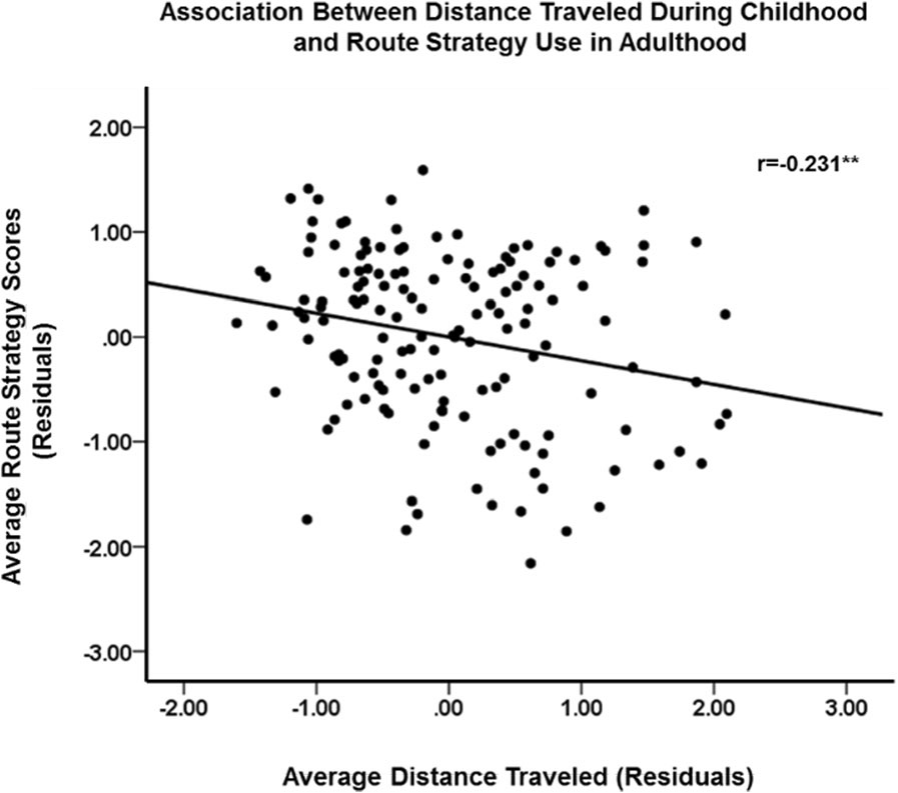
Individual differences in the relationship between distance traveled and route strategy use, controlling for participant sex and trait anxiety. **p < .01

Total childhood wayfinding experience negatively predicted route strategy, *B* = −0.223, *p* = .010, but did not predict orientation strategy, *B* = 0.003, *p* = .971. The effect of total wayfinding experience on route strategy remained significant when controlling only for participant sex, *B* = −0.179, *p* = .042 or trait anxiety, *B* = −0.206, *p* = .018, but became marginally significant when controlling for both participant sex and trait anxiety, *B* = −0.165, *p* = .060. This negative effect was found for females, *B* = −0.287, *p* = .005, but not males, *B* = −0.018, *p* = .906. When analyzing the two separate components of childhood wayfinding experience, distance traveled was more crucial in predicting their route strategy, *B* = −0.279, *p* < .001, than time spent outdoors, which was not significantly related to route strategy, *B* = −0.145, *p* = .069. Critically, distance traveled remained negatively related to route strategy, even when controlling for participant sex and trait anxiety, *B* = −0.226, *p* = .005 (see Fig. 6 for scatterplot of relationship between distance traveled and route strategy, controlling for participant sex and trait anxiety). Again, the negative effect of distance traveled on route strategy was present for females, *B* = −0.296, *p* = .001 but not males, *B* = −0.154, *p* = .307.

**Fig. 7:**
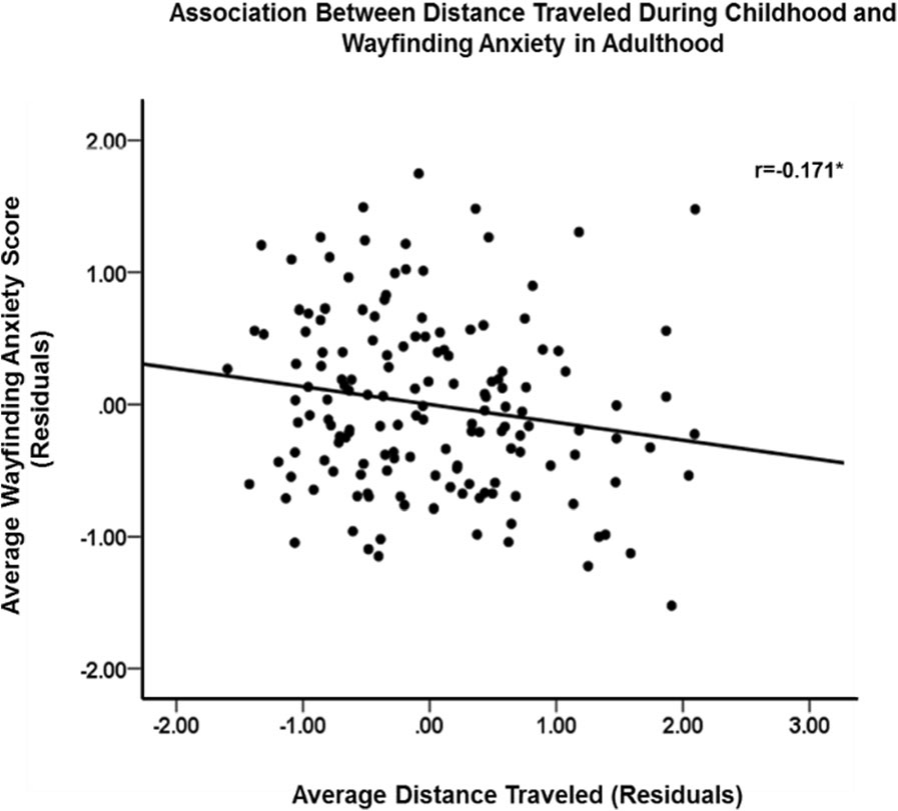
Individual differences in the relationship between distance traveled and wayfinding anxiety, controlling for participant sex and trait anxiety. *p < .05

Total childhood wayfinding experience negatively predicted wayfinding anxiety, *B* = −0.190; *p* = .009, whether controlling for participant sex, *B* = −0.150, *p* = .042 or trait anxiety, *B* = −0.157, *p* = .023, but not when controlling for both simultaneously, *B* = −0.124, *p* = .076. This negative effect controlling for trait anxiety was found for males, *B* = −0.253, *p* = .027, but not females, *B* = −0.046, *p* = .599. More specifically, distance traveled, *B* = −0.200, *p* = .003, but not time spent outdoors, *B* = 0.108, *p* = .107, negatively predicted participants’ wayfinding anxiety, even after controlling for participant sex and trait anxiety, *B* = −0.136, *p* = .036 (see Fig. 7 for scatterplot of relationship between distance traveled and wayfinding anxiety, controlling for participant sex and trait anxiety). Again, the relationship between distance traveled and wayfinding anxiety when controlling for trait anxiety was found for males, *B* = −0.246, *p* = .030, but not for females, *B* = −0.080, *p* = .303.

### Aim 4: Does wayfinding strategy explain individual differences in the relationship between childhood wayfinding experience and wayfinding anxiety?

#### Wayfinding strategy as mediator

Following Schug’s (2016b) proposal that navigational style, or what we call “wayfinding strategy” in the current study, might mediate the association between wayfinding experience and wayfinding anxiety, we first ran a mediation analyses with total childhood wayfinding experience and then ran follow-up analyses with specific types of childhood wayfinding experiences (distance traveled and time spent outside) while controlling for participant sex and trait anxiety. Because we were interested in examining individual differences, we controlled for participant sex.

When examining the role of wayfinding strategy on the relationship between total childhood wayfinding experience and wayfinding anxiety, we found that neither route strategy nor orientation strategy significantly mediated the relationship between total childhood wayfinding experience and wayfinding anxiety when controlling for participant sex and trait anxiety (see Supplementary Materials, Supplemental Figure 5a for null findings related to route strategy, and Supplemental Figure 6a-c for null findings related to orientation strategy).

**Fig. 8:**
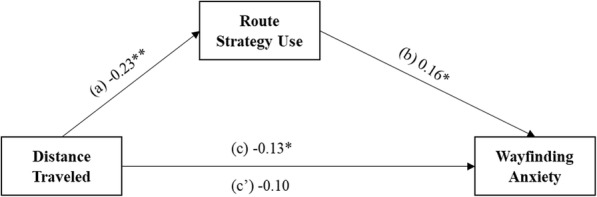
Direct and indirect effects of distance traveled on wayfinding anxiety through the mediator route strategy, controlling for participant sex and trait anxiety. *p < .05, **p < .01, ***p < .001

However, when we assessed type of childhood wayfinding experience, we found that route strategy mediated the relationship between the distance traveled (see Fig. 8 for mediation model), but not time spent outdoors (see Supplementary Materials, Supplemental Figure 5b, for null findings related to time spent outdoors), and wayfinding anxiety, controlling for participant sex and trait anxiety. The total effect of distance traveled on wayfinding anxiety, while controlling for trait anxiety, was statistically significant, path *c* = − 0.13, *t*(147) = − 2.06, *p* = .041, 95% CI [− 0.26, − 0.01], suggesting that participants who traveled farther distances as children have lower levels of wayfinding anxiety as adults when the effect of the mediator route strategy was left unaltered. The association between distance traveled and route strategy use, when controlling for participant sex and trait anxiety, was significant, path *a* = − 0.23, *t*(147) = − 2.88, *p* = .005, 95% CI [− 0.38, − 0.07]. The relationship between route strategy and wayfinding anxiety when controlling for distance traveled, participant sex, and trait anxiety was also statistically significant, path *b* = 0.16, *t*(146) = 2.37, *p* = .019, 95% CI [0.03, 0.29]. Finally, the direct effect of distance traveled on wayfinding anxiety, while controlling for route strategy, participant sex, and trait anxiety was no longer statistically significant, path *c′* = − 0.10, *t*(146) = − 1.49, *p* = .139, 95% CI [− 0.23, .03]. The negative coefficient of the direct effect indicates that participants who traveled farther distances as children had a 0.10 unit decrease in wayfinding anxiety when route strategy use, participant sex, and trait anxiety were held constant.

#### Wayfinding anxiety as mediator (reverse causal mediation)

Because anxiety can affect decision-making (Hartley & Phelps, 2012), we hypothesized that participants’ wayfinding anxiety might instead explain individual differences in the relationship between childhood wayfinding experience and wayfinding strategy. That is, we reasoned that the reverse causal mediation, whereby wayfinding anxiety mediates the relationship between childhood wayfinding experience and wayfinding strategy, was also a plausible developmental cascade. Again, we controlled for participant sex as well as trait anxiety.

**Fig. 9:**
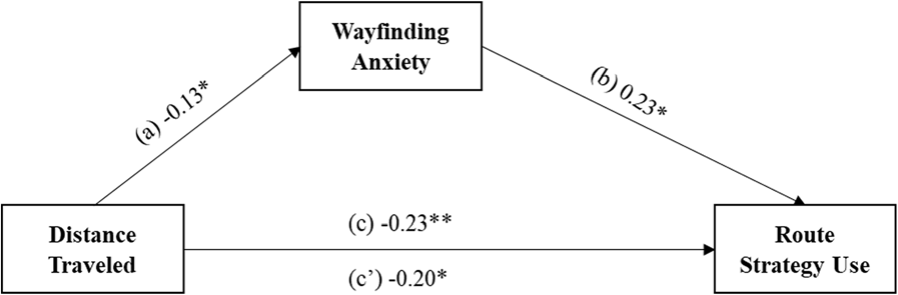
Direct and indirect effects of distance traveled on route strategy through the mediator wayfinding anxiety, controlling for participant sex and trait anxiety. *p < .05, **p < .01, ***p < .001

We found that wayfinding anxiety did not mediate the relationship between total childhood wayfinding experience and route strategy (see Supplementary Materials, Supplemental Figure 7a, for null findings related to route strategy) or orientation strategy (see Supplementary Materials, Supplemental Figure 8a-c, for null findings related to orientation strategy) when controlling for participant sex and trait anxiety. We also examined type of early childhood wayfinding experience and found that wayfinding anxiety partially mediated the relationship between distance traveled and route strategy use, controlling for participant sex and trait anxiety (see Fig. 9 for mediation model). We did not see a similar mediation effect with time spent outdoors, so we only report the analysis with distance traveled in the main text (see Supplementary Materials, Supplemental Figure 7b, for null findings related to time spent outdoors). The total effect of distance traveled on route strategy use when controlling for participant sex and trait anxiety was statistically significant, path *c* = − 0.23, *t*(147) = − 2.88, *p* = .005, 95% CI [− 0.38, − 0.07]. Participants who traveled farther distances as children currently used a route strategy less often when the effect of the mediator wayfinding anxiety was not held constant. Distance traveled was significantly related to wayfinding anxiety, controlling for participant sex and trait anxiety, path *a* = − 0.13, *t*(147) = − 2.06, *p* = .041, 95% CI [− 0.26, − 0.01]. Wayfinding anxiety and route strategy use controlling for distance traveled, participant sex, and trait anxiety was also significant, path *b* = 0.23, *t*(146) = 2.37, *p* = .019, 95% CI [0.04, 0.43]. The direct effect of distance traveled on route strategy controlling for wayfinding anxiety, participant sex, and trait anxiety remained significant, path *c′* = − 0.20, *t*(146) = − 2.49, *p* = .014, 95% CI [− 0.35, − 0.04]. Participants who traveled farther distances as children had a 0.20 unit decrease in route strategy use, when wayfinding anxiety, participant sex, and trait anxiety were held constant. Mediation analyses were corrected for multiple comparisons using an FDR procedure (Benjamini & Hochberg, 1995).

## Discussion

The current study sought to examine the effects of different aspects of adults’ early childhood wayfinding experience (i.e., distance traveled and time spent outside) on their current wayfinding strategies (i.e., route and orientation strategy) and levels of wayfinding anxiety, independent of their general anxiety levels. While other studies have examined childhood wayfinding experiences by when they occurred (i.e., younger vs. older age groups; e.g., Schug, 2016b), ours sought to assess the type of experience (distance traveled or time spent outdoors) that individuals reported. Furthermore, our study differs from previous studies investigating wayfinding strategies in that it gives special attention to route strategy use. For example, while most similar studies focus on how using an orientation strategy—the more efficient strategy for navigating—might relate to childhood wayfinding experience and/or later wayfinding anxiety (Lawton, 1994, 1996;Lawton & Kallai, 2002 ; Schug, 2016b), we examined both types of wayfinding approaches. Last, the current findings provide further support, as well as some nuanced explanations, for sex and individual differences in large-scale spatial behaviors, specifically wayfinding.

As we predicted (Aim 1), males reported greater freedom to roam their environments during childhood than did females. Males reported being allowed to travel farther distances and play outside more frequently alone or with friends than did females. We also saw sex differences in wayfinding strategy, with males reporting increased use of an orientation strategy and females reporting increased use of a route strategy, which is argued to be a more inefficient strategy by which to navigate (Lawton, 1996). Interestingly, both males and females in this study reported using a route strategy more often than an orientation strategy when traveling, which contradicts prior reported results in an American and Hungarian sample (Lawton & Kallai, 2002). While speculative, increased use of a route strategy among both males and females may be a product of recent technological advances. Perhaps the advent of smartphones and, thus, increased reliance on readily available GPS to navigate in the last decade may have allowed individuals to become more complacent when traveling to unfamiliar locations. As such, we may no longer feel the need to learn how to orient ourselves by “invisible” global references. Finally, we also found higher levels of wayfinding anxiety in females than in males, which was not explained by general (i.e., trait) anxiety, because there were no significant differences in trait anxiety between the sexes. Taken together, most of these findings replicate those found in prior literature (Lawton & Kallai, 2002; Schug, 2016b) and show significant, reliable sex differences in those factors (i.e., early childhood wayfinding experience, wayfinding strategy use, and wayfinding anxiety) that potentially explain later sex and individual differences in large-scale spatial abilities, such as navigation and wayfinding.

We also examined whether sex differences in wayfinding strategies and wayfinding anxiety could be explained by different childhood wayfinding experiences (Aim 2). We found that distance traveled (but not time spent outdoors) mediated the sex difference in route (but not orientation) strategy use. Our findings are similar to those of Lawton and Kallai (2002), who tested whether childhood wayfinding experience would mediate sex differences in orientation strategy preference but did not find such an effect. However, our study is unique in that we explored different types of childhood wayfinding experience, as well as the two wayfinding strategies in our mediation analyses. Our findings lend support to the idea that females develop a preference for a route strategy to navigate, in part, because they are given fewer opportunities to explore their outdoor environments, or, more specifically, travel farther distances on their own or with friends, as children. However, longitudinal studies are needed to assess the relationship between childhood experience with navigation and the development of wayfinding strategies in real time.

In keeping with the goals of Aim 2, we also found that when controlling for general anxiety, distance traveled during childhood (but not time spent outdoors) mediated the sex difference in wayfinding anxiety. This contrasts with findings reported by Lawton and Kallai (2002), who found no evidence for a mediation effect of childhood wayfinding experience on sex differences in wayfinding anxiety. One possible explanation for this is that wayfinding experience may not be the only factor affecting sex differences in wayfinding anxiety. For example, Lawton and Kallai (2002) found that individuals’ self-reported feelings of personal safety when navigating mediated the sex difference in wayfinding anxiety. While we did not measure feelings of personal safety, we did investigate whether the relationship between total childhood wayfinding experience (and then distance traveled) and wayfinding anxiety was found in both males and females when each group was analyzed separately. We found a significant negative relationship between the two variables in males but not females, lending support to the idea that, at least for females, fears about navigating unfamiliar areas may be driven by other concerns besides getting lost. Nevertheless, although ours is one of the only studies to assess mediating effects of childhood wayfinding experience on sex differences in wayfinding affect, our findings provide evidence that giving children more freedom to roam outdoors unmonitored may mitigate later apprehension about navigating.

Individual differences in participants’ early wayfinding experience did significantly relate to wayfinding strategy use, even after we controlled for participant sex and general anxiety separately (Aim 3). Contrary to previously published research (Lawton, 1996), only route strategy use was related to childhood wayfinding experience, especially distance traveled. Specifically, individuals who explored their outdoor surroundings more as children tended to use route information less often when navigating as adults, independent of their general anxiety levels. Given previous studies showing that children who are allowed to roam wider ranges have improved navigation abilities compared with children who are more restricted (for a review, see Schug, 2016a), it was not surprising that we found that individuals who traveled farther distances as children did not rely as much on the less efficient strategy for wayfinding. Interestingly, when looking at this relationship in males and females separately, we found that the negative effect was only present in females, possibly as a result of their lower amounts of childhood wayfinding experience and higher use of a route strategy than were seen with males. Furthermore, although we found an overall negative association between childhood wayfinding experience, particularly distance traveled, and route strategy use in adulthood, this does not necessarily suggest that individuals who were highly exploratory children, in turn, developed a predominantly orientation-based style of navigating, because we did not find a significant relationship between early wayfinding experience and orientation strategy use. One possible explanation for this finding is that the use of route versus orientation information for wayfinding may not be dichotomous, nor are the two strategies mutually exclusive. Perhaps some individuals learn how to navigate efficiently using another approach that does not require either route or orientation/survey knowledge, while others may use both strategies at roughly equal rates. Another possible explanation for the lack of a significant finding with orientation strategy use may have to do with the lower frequency with which our sample reportedly relied on orientation rather than route information for wayfinding compared with cross-cultural samples in previous studies (Lawton & Kallai, 2002; Schug, 2016b). On the other hand, Lawton and Kallai (2002) only found a small positive correlation between adults’ self-reported childhood wayfinding experience and their orientation strategy use. Thus, there is weak but inconclusive support for the idea that use of an orientation strategy develops as a function of navigating extensively during childhood. In sum, our novel findings suggest that (1) traveling farther distances does not mean children will learn to use orientation information when navigating, and (2) to prevent children from developing less efficient wayfinding strategies, it is not sufficient to allow them to play outside more; they must also be allowed to roam farther away from their homes (e.g., walking to school or a nearby friend’s house).

Because high levels of anxiety can negatively impact working memory (Vytal et al., 2013), we also sought to examine whether individual differences in early childhood wayfinding experience were related to later wayfinding anxiety, controlling for participant sex and general anxiety (Aim 3). Whereas Schug (2016b) found no conclusive link between self-reported childhood experience and later wayfinding anxiety, we found that, overall, individuals who had greater total wayfinding experience as children reported lower levels of anxiety about navigating as adults. Again, how far individuals traveled, but not amount of time spent outside, during childhood was negatively correlated with their current levels of wayfinding anxiety. This significant relationship persisted even after we controlled for participant sex and trait anxiety, although it was found in males but not females, indicating, once again, that females’ wayfinding anxiety may be influenced by factors other than wayfinding experience (e.g., feeling unsafe when navigating unfamiliar areas). Taken together, these findings suggest that allowing children not simply to play outdoors but to roam their neighborhoods might mitigate their apprehensive feelings about navigating later in life, but that for females, this relationship may be more complicated.

Finally, we looked at whether wayfinding strategy explained individual differences, regardless of participant sex and general anxiety, in the relationship between childhood wayfinding experience and wayfinding anxiety (Aim 4). We found that a preference for a route strategy rather than an orientation strategy was associated with experiential and affective factors related to wayfinding. Specifically, although others have proposed that an orientation style of navigating might mediate the relationship between wayfinding experience and anxiety (see Schug, 2016b), we found that orientation strategy use was related to neither childhood experience nor later wayfinding anxiety. However, route strategy preference was negatively related to both in our sample, indicating that the link between experience and later anxiety might be mediated by a preference for navigating by route information. Indeed, we found that adults’ use of a route strategy when navigating mediated the relationship between their childhood wayfinding experience and their current wayfinding anxiety, regardless of their sex or generalized anxiety. However, for the current study, we reasoned that anxiety when navigating might also affect individuals’ wayfinding strategy preferences, and, as a result, we conducted the reverse causal mediation analysis. Highly anxious individuals might opt to use route information such as salient features in the environment when traveling because such information is easier to fixate on and, thus, may allay their worries about getting lost. Therefore, we switched the mediator (route use) and outcome (wayfinding anxiety) and found that wayfinding anxiety also mediated the relationship between wayfinding experience, particularly distance traveled, and route strategy use when controlling for participant sex and general anxiety. Because these findings are correlational, the directionality between route strategy use and wayfinding anxiety remains unclear. We discuss one potential avenue for addressing the issue of causality in the next section.

### Limitations and future directions

Our study design was limited by the fact that the measures we examined were all based on self-report and were not independent assessments and observations. More objective behavioral measures (e.g., independent coders examining videos of children playing outdoors, parental assessments of children’s daily activities, virtual/real-world navigation tasks that test route and orientation knowledge) are needed to better evaluate the relationships between spatial experience, strategy use, and anxiety. Second, because this study is correlational as well as cross-sectional, we cannot make any definitive causal claims between the variables observed. However, we can attempt to make sensible claims about which variables we think might precede others. For example, we know from longitudinal studies that childhood experiences affect later outcomes (Schilling, Aseltine, & Gore, 2007; Thompson, Humbert, & Mirwald, 2003), so it made the most sense to hypothesize that scores related to childhood wayfinding experience between the ages of 6 and 15 years old would predict adult wayfinding strategy and wayfinding anxiety levels. However, we also recognize that individuals who are keener at navigating by more efficient strategies, regardless of how they learned those strategies, are more likely to choose to roam farther away from familiar areas. Furthermore, wayfinding anxiety may be learned from observing a caregiver’s negative reaction to getting lost rather than having little wayfinding experience. Nevertheless, given that only childhood wayfinding experience was assessed in retrospect, we reasoned that this factor was the most sensible predictor of concurrent wayfinding strategies and anxiety. Where we absolutely could not distinguish cause and effect was between variables related to current behaviors (i.e., wayfinding strategy and wayfinding anxiety). In said cases, we conducted double-mediation analyses, swapping the mediator (route strategy preference) and outcome (wayfinding anxiety) to assess whether the results held either way, which turned out to be the case.

We must keep in mind the possibility of bidirectional relationships between experience and ability if future studies are to disentangle these effects using longitudinal, experimental, and intervention designs. For example, one way to determine whether wayfinding strategy use influences wayfinding anxiety would be to conduct a study with an experimental design in which individuals are randomly assigned to use one type of wayfinding strategy over another (e.g., by placing them in an environment where landmark/route or geometric/orientation information is unavailable) and their wayfinding anxiety levels are subsequently assessed. Thus, future researchers may want to investigate early experiential factors concurrently and longitudinally to assess effects of these factors on later wayfinding outcomes more adequately.

## Conclusion

We sought to examine the effects of childhood wayfinding experience on later wayfinding strategy use and wayfinding anxiety, with the goal of explaining sex and individual differences in these behaviors. Sex differences in childhood wayfinding experience may help us understand why males and females develop different approaches to wayfinding in the environment and different levels of wayfinding anxiety. However, rather than directly predicting the development of a highly efficient strategy for navigating, environmental exploration during childhood may instead help children do away with inefficient strategies early on, as evidenced by the negative association between childhood wayfinding experience and route strategy use. Furthermore, the relationship between wayfinding anxiety and wayfinding strategy may be cyclical: Fear about becoming lost may influence the decision to use readily available yet unreliable route information when navigating, which may result in getting lost again and developing more wayfinding anxiety. Nevertheless, it is plausible that allowing children to widely explore their environments may lead them to develop less anxiety about navigating and perhaps develop successful wayfinding strategies, even if not strictly orientation-based. Importantly, the current findings related to wayfinding anxiety could not be attributed to participants’ generalized anxiety, suggesting that we should pay particular attention to domain-specific spatial anxiety when examining relationships between childhood spatial experience and later spatial strategy use, affect, and ability. Finally, our results suggest that sex and individual differences in spatial reasoning may be the indirect result of differences in childhood experiences that lead to differences in both affect and approaches or strategies related to spatial tasks.

## Supplementary information

**Additional file 1: Supplemental Figure 1.** Direct and indirect effects of *Participant Sex* on *Orientation Strategy Use* through the mediator, *Total Childhood Wayfinding Experience* (a), *Time Spent Outdoors* (b), and *Distance Traveled* (c), controlling for *Trait Anxiety*. *Note.* *p < 0.05, **p < 0.01. **Supplemental Figure 2.** Direct and indirect effects of *Participant Sex* on *Route Strategy Use* through the mediator, *Time Spent Outdoors*, controlling for *Trait Anxiety*. *Note.* *p < 0.05, **p < 0.01. **Supplemental Figure 3.** Direct and indirect effects of *Participant Sex* on *Route Strategy Use* through the mediator, *Distance Traveled*, controlling for *Wayfinding Anxiety*. *Note.* *p < 0.05. **Supplemental Figure 4.** Direct and indirect effects of *Participant Sex* on *Wayfinding Anxiety* through the mediator, *Total Childhood Wayfinding Experience* (a) and *Time Spent Outdoors* (b), controlling for *Trait Anxiety*. *Note.* *p < 0.05, **p < 0.01. **Supplemental Figure 5.** Direct and indirect effects of *Total Childhood Wayfinding Experience* (a) and *Time Spent Outdoors* (b) on *Wayfinding Anxiety* through the mediator, *Route Strategy*, controlling for *Participant Sex* and *Trait Anxiety*. *Note.* *p < 0.05. **Supplemental Figure 6.** Direct and indirect effects of *Total Childhood Wayfinding Experience* (a), *Time Spent Outdoors* (b), and *Distance Traveled* (c) on *Wayfinding Anxiety* through the mediator, *Orientation Strategy*, controlling for *Participant Sex* and *Trait Anxiety*. *Note.* *p < 0.05. **Supplemental Figure 7.** Direct and indirect effects of *Total Childhood Wayfinding Experience* (a) and Time Spent Outdoors (b) on *Route Strategy* through the mediator, *Wayfinding Anxiety*, controlling for *Participant Sex* and *Trait Anxiety*. *Note.* *p < 0.05. **Supplemental Figure 8.** Direct and indirect effects of *Total Childhood Wayfinding Experience* (a), Time Spent Outdoors (b), and Distance Traveled (c) on *Orientation Strategy Use* through the mediator, *Wayfinding Anxiety*, controlling for *Participant Sex* and *Trait Anxiety*. *Note.* *p < 0.05.

## Data Availability

Data and materials are available upon request from the first author.

## Abbreviations

GPS: Global Positioning System
STAI: State-Trait Anxiety Inventory
