# Supplementary material for: Childhood wayfinding experience explains sex and individual differences in adult wayfinding strategy and anxiety
Source: Cogn Res Princ Implic. 2020 Mar 17;5:12. doi: 10.1186/s41235-020-00220-x (PMC7078395; doi:10.1186/s41235-020-00220-x)
Supplement: Supplementary file 1 — Additional file 1: Supplemental Figure 1. Direct and indirect effects of Participant Sex on Orientation Strategy Use through the mediator, Total Childhood Wayfinding Experience (a), Time Spent Outdoors (b), and Distance Traveled (c), controlling for Trait Anxiety. Note. *p < 0.05, **p < 0.01. Supplemental Figure 2. Direct and indirect effects of Participant Sex on Route Strategy Use through the mediator, Time Spent Outdoors, controlling for Trait Anxiety. Note. *p < 0.05, **p < 0.01. Supplemental Figure 3. Direct and indirect effects of Participant Sex on Route Strategy Use through the mediator, Distance Traveled, controlling for Wayfinding Anxiety. Note. *p < 0.05. Supplemental Figure 4. Direct and indirect effects of Participant Sex on Wayfinding Anxiety through the mediator, Total Childhood Wayfinding Experience (a) and Time Spent Outdoors (b), controlling for Trait Anxiety. Note. *p < 0.05, **p < 0.01. Supplemental Figure 5. Direct and indirect effects of Total Childhood Wayfinding Experience (a) and Time Spent Outdoors (b) on Wayfinding Anxiety through the mediator, Route Strategy, controlling for Participant Sex and Trait Anxiety. Note. *p < 0.05. Supplemental Figure 6. Direct and indirect effects of Total Childhood Wayfinding Experience (a), Time Spent Outdoors (b), and Distance Traveled (c) on Wayfinding Anxiety through the mediator, Orientation Strategy, controlling for Participant Sex and Trait Anxiety. Note. *p < 0.05. Supplemental Figure 7. Direct and indirect effects of Total Childhood Wayfinding Experience (a) and Time Spent Outdoors (b) on Route Strategy through the mediator, Wayfinding Anxiety, controlling for Participant Sex and Trait Anxiety. Note. *p < 0.05. Supplemental Figure 8. Direct and indirect effects of Total Childhood Wayfinding Experience (a), Time Spent Outdoors (b), and Distance Traveled (c) on Orientation Strategy Use through the mediator, Wayfinding Anxiety, controlling for Participant Sex and Trait Anxiety. Note. *p < 0.05. [file 41235_2020_220_MOESM1_ESM.docx]

**
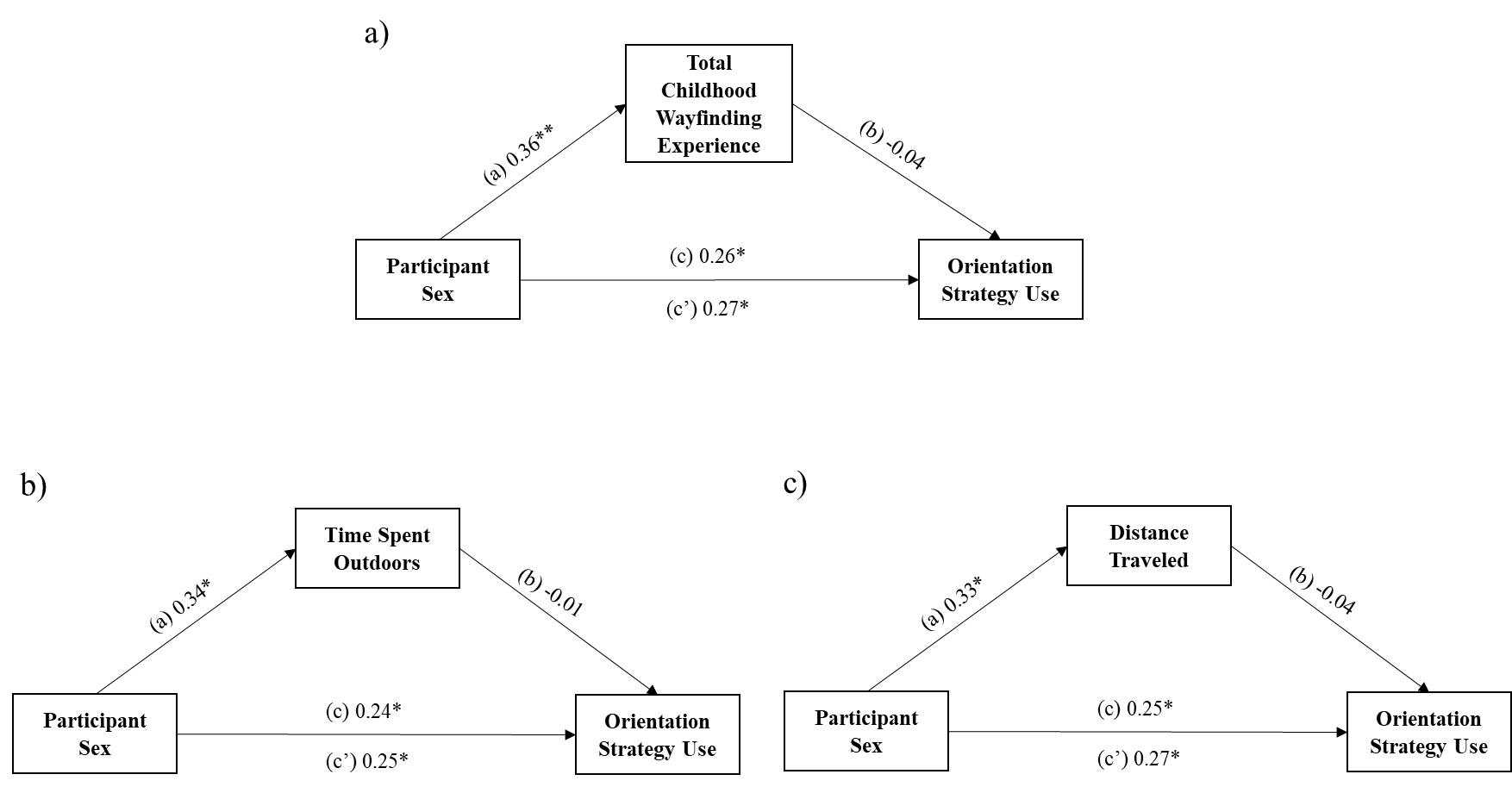
Supplementary Materials**

**Supplemental Figure 1.** Direct and indirect effects of *Participant Sex* on *Orientation Strategy Use* through the mediator, *Total Childhood Wayfinding Experience* (a), *Time Spent Outdoors* (b), and *Distance Traveled* (c), controlling for *Trait Anxiety*. *Note.* **p* < 0.05, ***p* < 0.01


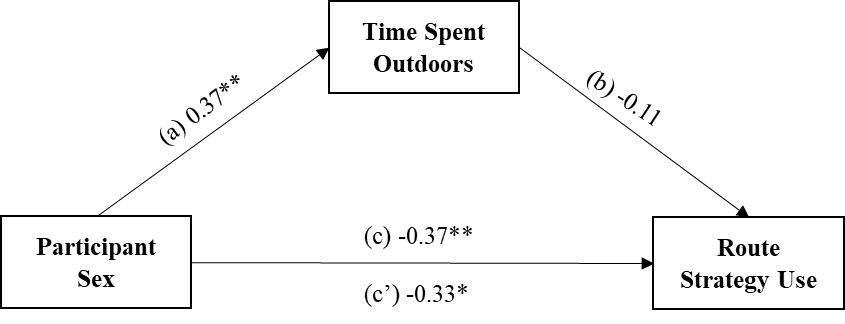


**Supplemental Figure 2.** Direct and indirect effects of *Participant Sex* on *Route Strategy Use* through the mediator, *Time Spent Outdoors*, controlling for *Trait Anxiety*. *Note.* **p* < 0.05, ***p* < 0.01

**Distance Traveled**

**Route Strategy Use**

(a) 0.25

(b) -0.20*

(c) -0.27*

(c’) -0.22

**Participant Sex**

**Supplemental Figure 3.** Direct and indirect effects of *Participant Sex* on *Route Strategy Use* through the mediator, *Distance Traveled*, controlling for *Wayfinding Anxiety*. *Note.* **p* < 0.05.


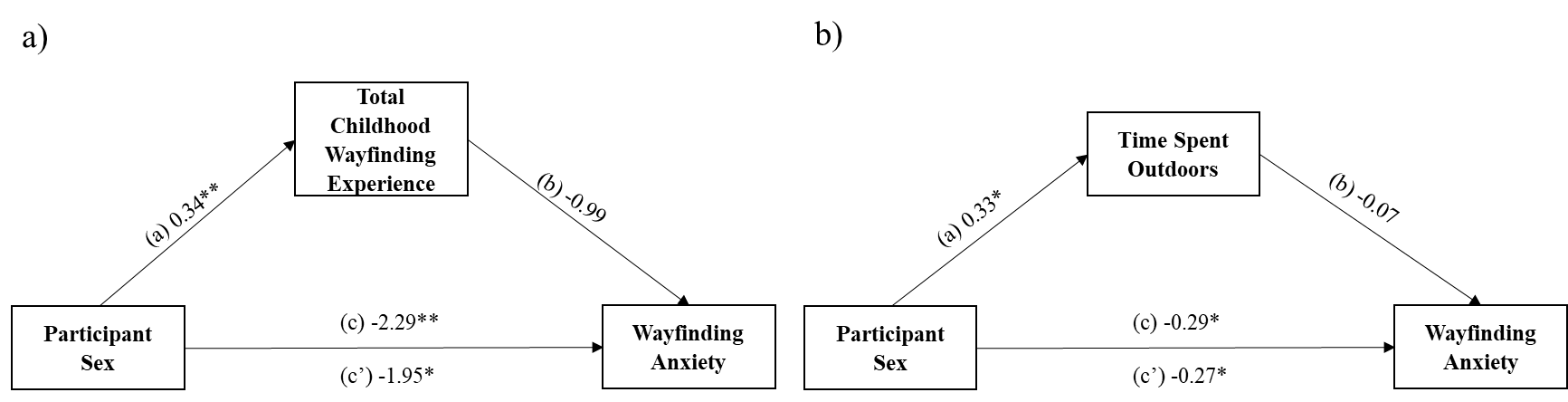


**Supplemental Figure 4.** Direct and indirect effects of *Participant Sex* on *Wayfinding Anxiety* through the mediator, *Total Childhood Wayfinding Experience* (a) and *Time Spent Outdoors* (b), controlling for *Trait Anxiety*. *Note.* **p* < 0.05, ***p* < 0.01

**
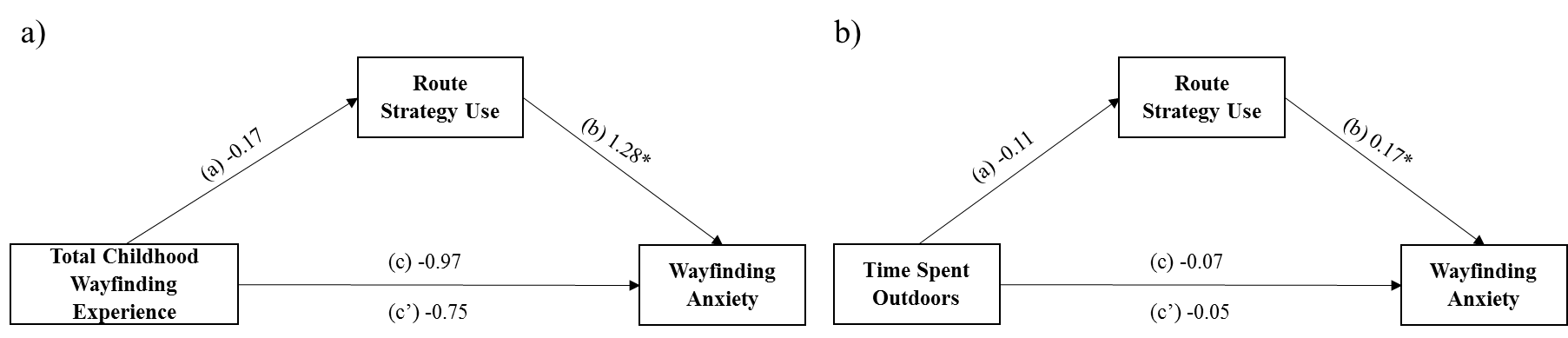
**

**Supplemental Figure 5.** Direct and indirect effects of *Total Childhood Wayfinding Experience* (a) and *Time Spent Outdoors* (b) on *Wayfinding Anxiety* through the mediator, *Route Strategy*, controlling for *Participant Sex* and *Trait Anxiety*. *Note.* **p* < 0.05


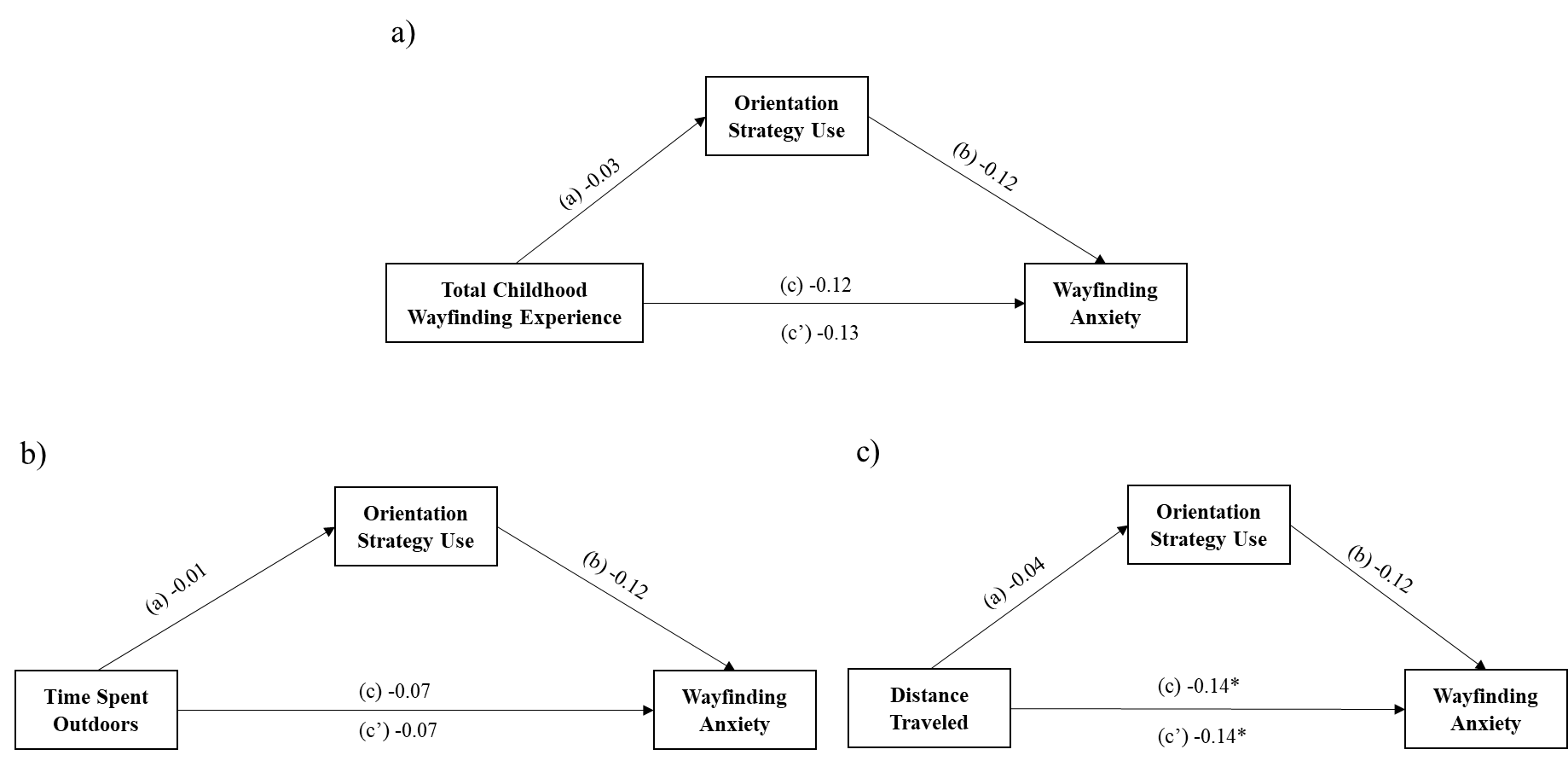


**Supplemental Figure 6.** Direct and indirect effects of *Total Childhood Wayfinding Experience* (a), *Time Spent Outdoors* (b), and *Distance Traveled* (c) on *Wayfinding Anxiety* through the mediator, *Orientation Strategy*, controlling for *Participant Sex* and *Trait Anxiety*. *Note.* **p* < 0.05


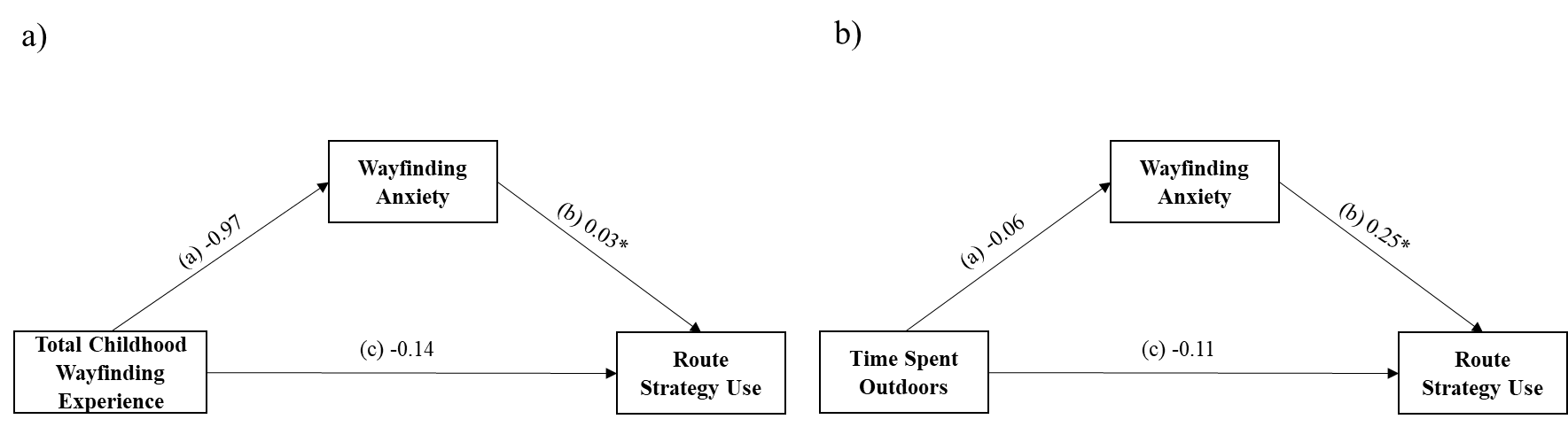


**Supplemental Figure 7.** Direct and indirect effects of *Total Childhood Wayfinding Experience* (a) and Time Spent Outdoors (b) on *Route Strategy* through the mediator, *Wayfinding Anxiety*, controlling for *Participant Sex* and *Trait Anxiety*. *Note.* **p* < 0.05


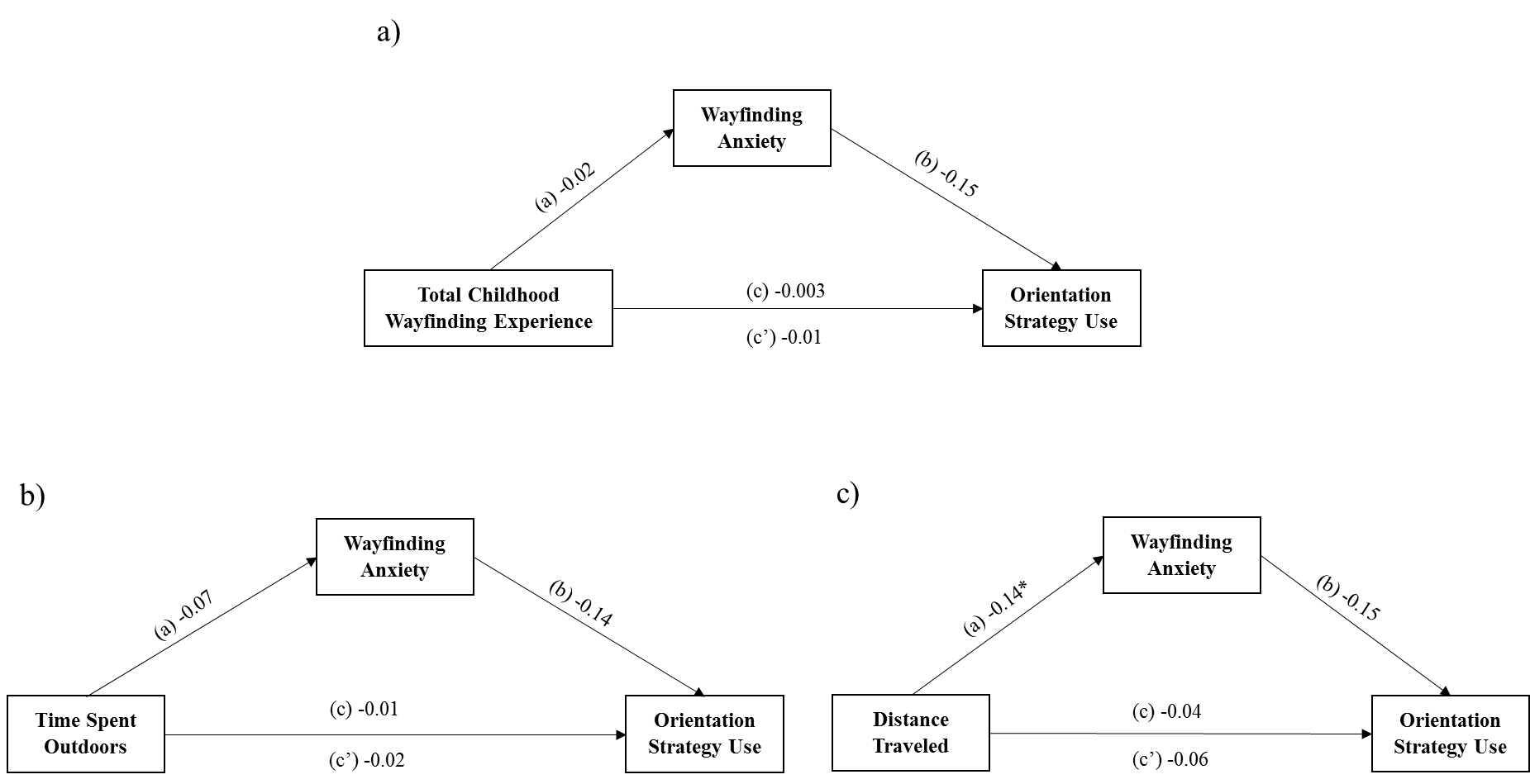


**Supplemental Figure 8.** Direct and indirect effects of *Total Childhood Wayfinding Experience* (a), Time Spent Outdoors (b), and Distance Traveled (c) on *Orientation Strategy Use* through the mediator, *Wayfinding Anxiety*, controlling for *Participant Sex* and *Trait Anxiety*. *Note.* **p* < 0.05
